# Intraneural ganglion cyst involving the tibial nerve—a case report

**DOI:** 10.1259/bjrcr.20160116

**Published:** 2017-01-25

**Authors:** Christina E Buckley, Emma Tong, Liam D Spence, Michael O’Shaughnessy

**Affiliations:** ^1^Plastic Surgery Unit, Cork University Hospital, Cork, Ireland; ^2^Department of Radiology, Cork University Hospital, Cork, Ireland

## Abstract

Intraneural ganglia are rare non-neoplastic cysts that are caused by an accumulation of thick mucinous fluid. This occurs within the epineurium of peripheral nerves, which is encased in a dense fibrous capsule. The most common presentation of this tumour is local and/or radiating pain. Involvement of the tibial nerve is extremely uncommon, with less than 18 reported cases in the literature. We present a case of an intraneural tibial nerve ganglion cyst in a young male. We also discuss the current literature and proposed pathogenesis and treatment of this rare entity.

## Clinical presentation

A 39-year-old machine operator presented with a 10-month history of pain, paresthesia and difficulty with dorsiflexion of the left foot. The pain initially commenced in his left hip, and then extended to the level of his left knee and calf after a further 2 and 5 months, respectively. Symptoms involved the plantar aspect of his left foot and hallux shortly thereafter. Cycling aggravated his symptoms. On examination, he was unable to flex his left hallux at the inter-phalangeal joint. He had reduced sensation on the plantar aspect of his first to fourth toes. Of note, he had no muscle wasting.

## Differential diagnosis

1. Peripheral nerve sheath tumour
2. Intraneural ganglion

### Imaging findings

**Figure 1.**
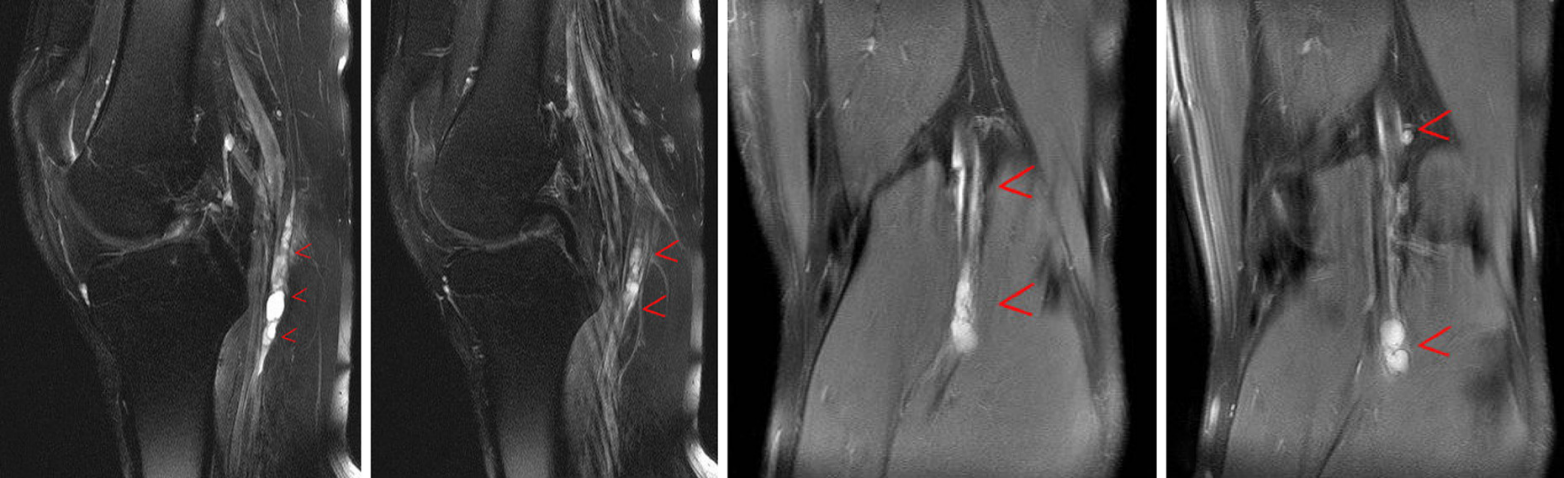
Sagittal and coronal MRI images of the left knee demonstrate a 9.2-cm multilobulated lesion with a vertical orientation in the vicinity of the proximal tibial nerve commencing at the popliteal fossa as highlighted by the red arrows (*T*_2 _turbo spin echo fat-saturated images).

**Figure 2.**
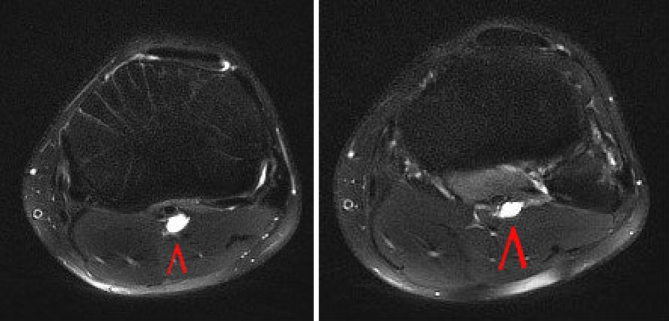
Axial MRI images of the left knee display peripheral enhancement of the tubular cystic mass involving the tibial nerve within its sheath extending from the popliteal fossa to the gastrocnemius muscle bellies, (red arrow).

MRI of the left knee demonstrated a multilobulated lesion with a vertical orientation in the vicinity of the proximal tibial nerve commencing at the popliteal fossa; this was 9.2 cm in length—as seen on the sagittal and coronal *T*_2 _turbo spin echo fat-saturated images (Figure 1). Axial images display peripheral enhancement of the tubular cystic mass involving the tibial nerve within its sheath extending from the popliteal fossa to the gastrocnemius muscle bellies (Figure 2). The constellation of findings was believed to represent a multilobulated ganglion and was likely contiguous with the posterior joint capsule.

## Treatment

**Figure 3.**
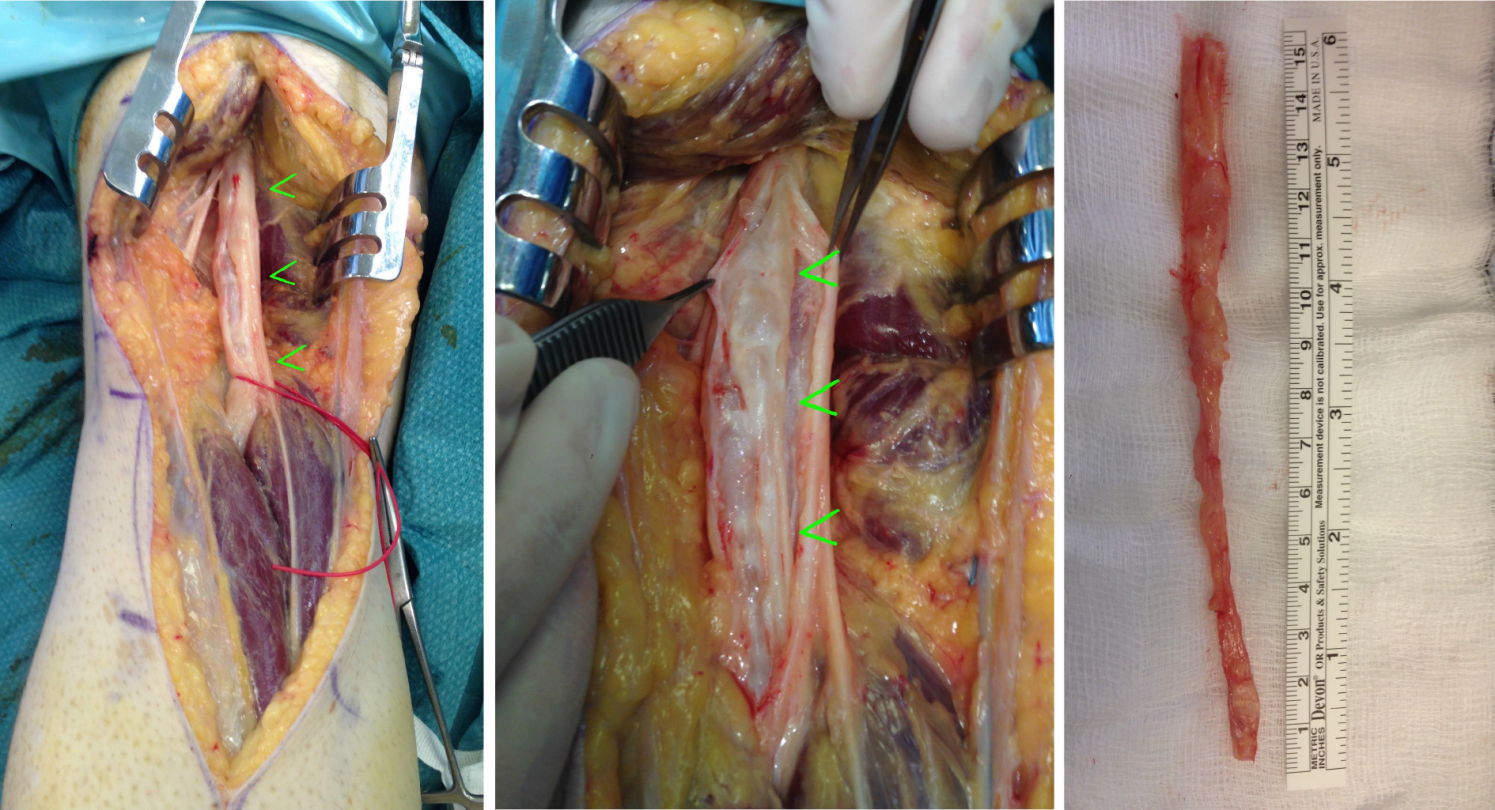
Intraoperative images showing surgical exploration of the popliteal fossa, revealing a firm multiloculated mass within the nerve sheath from the proximal end of the popliteal fossa deep to the lateral gastrocnemius muscle belly origin over a length of 22 cm. Here one can see how the tumour is intimately related to the tibial nerve, and the postoperative specimen sent for pathology review.

Surgical exploration of the posterior leg revealed a firm multiloculated mass within the nerve sheath from the proximal end of the popliteal fossa deep to the lateral gastrocnemius muscle belly origin over a length of 22 cm. It was intimately related to the tibial nerve over this length making the dissection challenging. The nerve was preserved throughout. During dissection from the epineural sheath, a portion of the mass was entered and the typical gelatinous fluid of a ganglion cyst was encountered (Figure 3). There was no apparent communication with the knee joint, but the distal end passed deep to the origin of the lateral gastrocnemius muscle belly—heading in the direction of the proximal tibiofibular joint. Histology revealed a fibrous walled cyst measuring 15.5 cm in length. The apparent lining of the cyst was not true epithelial or endothelial lining, which was consistent with a pseudocyst or ganglion.

## Outcome

Postoperatively the patient’s pain resolved within the first 2 weeks. His paresthesia and weakness improved gradually over 6 months. Following postoperative rehabilitation with physiotherapy and scar therapy, he is currently asymptomatic and has no sensory or motor dysfunction.

## Discussion

Ganglion cysts are mucin-filled structures associated with joint capsules, tendons or tendon sheaths. Intraneural ganglia are rare non-neoplastic cysts that are caused by an accumulation of thick mucinous fluid. This occurs within the epineurium of peripheral nerves, which is encased in a dense fibrous capsule. If these cysts grow large enough to cause compression of adjacent nerve fascicles, this can lead to pain, paraesthesia, weakness, muscle denervation and atrophy. The most common presentation of this tumour is local and/or radiating pain. However, there have also been cases reporting sensory and motor deficits.

Intraneural ganglion cysts uncommonly occur in the vicinity of peripheral nerves. They most commonly present in the dorsal carpal region but their location is variable. The radial, ulnar, median, sciatic, tibial and posterior interosseous nerves are other reported sites. Involvement of the tibial nerve is exceptionally rare, with less than 15 reported cases in the literature. The first case of intraneural ganglion cyst of the tibial nerve was described in 1967. Since then, 17 cases have been reported.^1–4^

The differential considerations for cystic intraneural lesions include cystic nerve sheath tumours, atypical Baker’s cyst and extraneural ganglion. MRI can differentiate cystic nerve sheath tumours such as Schwannomas and extraneural ganglia from cystic intraneural lesions. A Baker’s cyst has a mass-like appearance, and is located extending from the tibiofemoral joint to within the confines of the medial head of the gastrocnemius and semi-membranous tendon insertion.^1^

The aetiology of these cystic structures is presumed to be secondary to trauma and synovial herniation, but remains unclear. Numerous hypotheses have been proposed for the pathogenesis of intraneural ganglia. Some of the earlier literature describe a degenerative process of connective and perineural tissue secondary to chronic mechanical “wear and tear.” Intraneural haemorrhage following trauma and the resultant formation of cysts after resorption of the bleed have also been proposed. However, this explanation is not convincing as the presence of haemosiderin deposits has been an infrequent finding. Trauma has also been implicated as a potential cause of this tumour formation, with several of the case reports indicating a history of trauma, albeit remote. Adn et al feel that trauma is more of a contributing factor rather than a causative factor. A new description based on analysis of clinical, operative and radiological findings has favoured a synovial origin of these intraneural ganglion cysts. This proposes that an articular nerve branch from a joint can act as the conduit for synovial fluid to leave the joint, which may be degenerative or traumatized. This was deemed the “unifying articular theory,” which was originally suggested for the intraneural ganglion cyst arising within the peroneal nerve. This was then later modified to include the tibial nerve as well.^5^

Definitive treatment consists of surgical excision, with preoperative planning aided by MRI. The most recent treatment option entails decompressing the intraneural ganglion cyst, followed by disconnecting the articular branch and then resecting the synovium. Partial cyst persistence, or cyst recurrence is most often due to incompletely disconnecting the articular branch. Similarly, inadequate resection of the synovium can lead to extraneural occurrences.^5^ New literature has described minimally invasive decompression techniques for the treatment of patients with intraneural ganglion cysts of the tibial nerve, the goal being to reduce nerve compression and secondary muscle denervation in patients wanting to avoid an open surgical approach. However, long-term follow-up studies are needed to determine cyst recurrence.^3^

## Learning points

1. This case illustrates the rare involvement of the tibial nerve in a cystic intraneural lesion.
2. The differential considerations for cystic intraneural lesions include cystic nerve sheath tumours, atypical Baker’s cyst and extraneural ganglion.
3. MRI can differentiate cystic nerve sheath tumours such as Schwannomas and extraneural ganglia from cystic intraneural lesions.
4. Definitive treatment consists of surgical excision, with preoperative planning aided by MRI.

## Consent

Written informed consent for the case to be published (including images, case history and data) was obtained from the patient(s) for publication of this case report, including accompanying images.
